# Serum uric acid and high-sensitivity C-reactive protein levels among people living with HIV on dolutegravir and ritonavir-boosted atazanavir-based antiretroviral therapy: a comparative cross-sectional study

**DOI:** 10.3389/fmed.2024.1370725

**Published:** 2024-07-17

**Authors:** Nuredin Chura Waritu, Suresh Kumar P. Nair, Rashed Edris Usure, Mohammed Jemal

**Affiliations:** ^1^Department of Biomedical Sciences, School of Medicine, Wolaita Sodo University, Wolaita Sodo, Ethiopia; ^2^Department of Biomedical Sciences, School of Medicine, Jimma University, Jimma, Ethiopia; ^3^Department of Pharmaceutical Chemistry, School of Pharmacy, Hawassa University, Hawassa, Ethiopia; ^4^Department of Biomedical Sciences, School of Medicine, Debre Markos University, Debre Markos, Ethiopia

**Keywords:** hyperuricemia, high-sensitivity C-reactive protein, dolutegravir, ritonavir-boosted atazanavir, HIV

## Abstract

**Background:**

After the introduction of antiretroviral therapy, the care given to people living with HIV has become complicated by the appearance of comorbidities as a result of HIV and HAART toxicities, in which cardiovascular disease got the most attention. So, this study aimed to assess serum uric acid and high-sensitivity C-reactive protein levels among people living with HIV on dolutegravir (DTG) and ritonavir-boosted atazanavir (ATV/r)-based therapy.

**Methods:**

An institutional-based comparative cross-sectional study was conducted from November 4, 2021, to January 4, 2022. An equal number of dolutegravir- and ritonavir-boosted atazanavir-treated patients (*n* = 86 each) were enrolled. A consecutive sampling method was used to select participants. Data were entered into Epidata version 4.6, exported to SPSS version 25.0, and analyzed using Chi-square, Student’s t-test, Mann–Whitney *U*-test, and logistic regression. Statistical significance was set at *p* < 0.05.

**Results:**

The prevalence of hyperuricemia and high-sensitivity C-reactive protein levels ≥2 mg/L were 46.5% (40/86) and 24.4% (21/86) in the DTG group, and 30.2% (26/86) and 44.2 (38/86) in the ATV/r group, respectively. When compared to ATV/r, a higher mean level of uric acid was found among DTG-based regimens (5.38 mg/dL). Duration of ART (AOR = 2, 95% CI: 1.2, 4.4) and DTG-based regimen (AOR = 1.9, 95% CI: 1.04, 3.8) were significant predictors of developing hyperuricemia. ATV/r-based regimen (AOR = 3, 95% CI: 1.5, 8.3) and high waist circumference (AOR = 2.5, 95% CI: 1, 3.5) were significantly associated with increased high-sensitivity C-reactive protein levels.

**Conclusion:**

It is observed that DTG-based and ATV/r-based ART are associated with hyperuricemia and increased high-sensitivity C-reactive protein levels, respectively. Therefore, it is important to consider and evaluate serum uric acid and high-sensitivity C-reactive protein levels in patients taking DTG and ATV/r-based ART, as well as among those on HAART for years and with a higher waist circumference, so as to detect and prevent early the risk of having CVD.

## Background

After the introduction of antiretroviral therapy (ART), the world successfully achieved higher viral suppression and a lower viral load burden, followed by a reduction in mortality and morbidity associated with HIV and an improvement in the quality of life of people living with HIV (1, 2). But, now a days, the care given to this population is complicated by the emergency of other comorbidities introduced by HIV itself and as a result of ART toxicities (3, 4). Exposure to ART for a longer duration of time enhances the susceptibility of these patients to developing these comorbidities, in which atherosclerotic cardiovascular disease (ASCVD) gains the most attention due to its association with the morbidity and mortality of people living with HIV (5–8).

Several studies have revealed that hyperuricemia and chronic inflammation have been proposed as potential mechanisms through which HIV and ART cause ASCVD (9, 10). Uric acid, the end product of purine metabolism, has been associated with metabolic syndrome, and it induces endothelial dysfunction, both of which are the main risk factors for ASCVD in general as well as in people living with HIV (9–16). Increased the activity of the pentose phosphate pathway (PPP) and increase the production of ribose 5-phosphate which stimulate purine nucleotide phosphorylase for nucleotide synthesis, that in turn increased the production of uric acid as well as ART-induced mitochondrial toxicity subsequently increased lactate which compete with uric to be excreted were the possible ways for hyperuricemia seen in treated HIV patients (17–19).

Hyperuricemia was increasingly seen among people living with HIV on ART than in the general population, which indicates people living with HIV are at greater risk of cardiovascular disease (CVD) than HIV-negative people, which means attention must be given to these people to prevent CVD (20). An increased risk of having hyperuricemia was seen among treatment-naive compared to treatment experienced people living with HIV (21, 22). It is observed that hyperuricemia was also detected in HIV patients taking HAART (23, 24). Accordingly, a study from Italy reported that 25.2% of people living with HIV on HAART developed elevated serum uric acid levels (12). The potential to cause an altered level of uric acid was different for different ART classes. Patients treated with stavudine, didanosine, and protease inhibitors had significantly increased levels of uric acid. However, tenofovir is associated with reduced levels of uric acid, and abacavir has a neutral effect on uric acid concentration (25).

Studies have reported that patients treated with dolutegravir (DTG) have elevated levels of uric acid (26, 27). Another study also showed people living with HIV on ritonavir-boosted protease inhibitors, especially ritonavir-boosted atazanavir (ATV/r), were observed to be hyperuricemic (28, 29).

Research into CVD risk factors has identified a number of inflammatory biomarkers, such as interleukin 6 (IL-6) and high-sensitivity C-reactive protein (hsCRP) (30–32). A study from Italy showed an increased hsCRP level was detected in people living with HIV on combination antiretroviral therapy (cART), which makes hsCRP a useful additional biomarker to predict the risk of HIV patients developing CVD (33, 34). The risk of elevated hsCRP levels was higher among people living with HIV than HIV-negative populations, but the risk was higher for treatment-naïve patients (35, 36). Another study showed similar hsCRP levels were observed during pre-ART and 36 weeks after commencing ART (37). However, one study from Ghana reported that 32.5% of patients on ART have increased hsCRP levels greater than or equal to 3 mg/L (31). A study from France complains that the use of ATV/r containing ART enhances inflammation due to its effect on activation of NF-kB and increased levels of different inflammatory biomarkers (38). It is speculated that the use of integrase strand transfer inhibitors (INSTI) is associated with reduced inflammation, which is determined by elevated levels of different inflammatory biomarkers including hsCRP levels (39). Inflammation as measured by hsCRP levels is reduced after patients switch to INSTI from boosted-protease inhibitors (40, 41). However, a conflicting result was posted, which indicates the association of INSTI including DTG with increased inflammation (42–44).

Several factors contributed to hyperuricemia and increased inflammation measured by hsCRP, such as duration of HIV and HAART, age, obesity, alcohol intake, smoking, region, ethnicity, and low CD4 cell count (45–47).

The DTG is a new integrase inhibitor that has been recommended by the World Health Organization (WHO) as the preferred first-line regimen for people living with HIV (48). ATV/r is widely used as a second-line therapy in Ethiopia (49). DTG is effective, has high antiviral potency, and can be used without pharmacological enhancements (50, 51). As a result of this, most of the patients are on a regimen containing these ARTs.

Despite this, there is no well-documented evidence that shows the level of uric acid and hsCRP among patients taking DTG and ATV/r-based regimens in sub-Saharan Africa, including Ethiopia. The current WHO guideline does not include uric acid monitoring in patients taking these regimens, but they are useful biomarkers to detect and prevent the early occurrence of CVD. Therefore, the present study is designed to assess the serum level of uric acid, hsCRP, and their associated factors among adult HIV patients taking DTG and ATV/r-based antiretroviral therapy at Jimma University Medical Center, Southwest Ethiopia.

## Materials and methods

### Study design and setting

An institutional-based comparative cross-sectional study was carried out at the ART clinic of Jimma University Medical Center (JUMC). The medical center has active 3,500 people living with HIV (until October 15, 2021); among these, 1841 were on ART containing DTG + 3TC + TDF, and 167 were on ART containing ATV/r + 3TC + TDF. The center provides services to the surrounding villages and nearby towns. Currently, the JUMC ART clinic provides voluntary counseling and testing (VCT), prevention of mother-to-child transmission of HIV, follow-up services for HIV-infected patients on ART therapy, and treatment of opportunistic infections.

### Study participant and study processes

The study was conducted from November 4, 2021, up to January 4, 2022. All people living with HIV ≥18 years who had received DTG-based and ATV/r-based regimens for at least 6 months (52) and who volunteered to participate in the study were included. Whereas, patients taking DTG-based and ATV/r-based ART regimens for less than 6 months, pre-existing liver and renal problems, or active cancer, and those who did not volunteer to participate in the study were excluded.

### Sample size determinations and sampling procedure

The number of participants included in the study was calculated using the G* power statistical power analysis version 3.1 software. The sample size was calculated by considering *α* = 0.05, power (1–β) = 90%, with a DTG to ATV/r ratio of 1:1, two tail *t*-tests, and an effect size of 0.5. The computed sample size was 172. Of these, 86 were in the DTG-based group and 86 were in the ATV/r-based group. A consecutive sampling technique was applied. All participants who fulfilled the inclusion criteria and were ready to participate were included in the study until the required sample size of 172 was achieved.

### Data collection procedures

Before data collection, training was provided to the data collectors for 1 day by the principal investigator. Data collection was controlled by the principal investigator, and the collected data were checked for completeness, consistency, and clarity. Trained nurses collected sociodemographic and related clinical data using a structured questionnaire. The time of initiation of HAART, medical history, WHO clinical stage, and viral load values were obtained from the medical record card. A sample of venous blood was collected from each study participant for laboratory testing by laboratory technologists. Anthropometric measurements such as height, weight, waist circumference, and hip circumference were collected by trained nurses using a standard balance and a SECA meter. BMI was calculated by dividing the individual’s weight in kilograms by the square of their height in meters (kg/m^2^). BMI was categorized as obese if BMI ≥25 kg/m^2^ and normal if BMI <25 kg/m^2^. The range of abnormal waist circumference for males and females was >102 and >88 cm, respectively. The cut-off for the waist-hip ratio was ≥0.9 for males and ≥0.85 for females, according to the criteria of the WHO guidelines (53, 54).

### Blood sample collection

To determine the serum UA, hsCRP, and CD4 cell count, 2 mL of blood was collected. Before centrifugation, whole blood was used to measure the CD4 cell count. The remaining blood was centrifuged at 3,000 rpm for 5 min. The serum obtained was stored at 4°C until the analysis of UA and hsCRP levels. UA and hsCRP levels were determined using a Roche Cobas 6,000 analyzer. The CD4 cell count was determined using a near-patient CD4 count system, which consisted of a FACSPresto counter machine and a BD FACSPresto™ Cartridge kit.

### Definitions

Hyperuricemia: patients having serum uric acid levels >6.3 mg/dL for males and >4.9 mg/dL for females (55).

Increased serum hsCRP levels: patients having serum hsCRP levels ≥2 mg/dL for both genders. It was categorized based on its risk enhancing factor for ASCVD (56).

Viral load (VL): ≤1,000 copies/ml was categorized as suppressed and >1,000 copies/ml as non-suppressed VL (57).

Patient on DTG-based regimen: patient taking DTG + 3TC + TDF for at least 6 months.

Patient on ATV/r-based regimen: patient taking ATV/r + 3TC + TDF for at least 6 months.

CD4 cell count: ≥500 cells/mm^3^ as normal range and competent immune system and <500 cells/mm^3^ as compromised immune system (58).

### Statistical methods

The data were checked, cleaned, entered into Epi-data software version 3.1, and exported to SPSS version 25 for further analysis. Simple descriptive statistics were used to present the participants’ sociodemographic characteristics. Categorical variables were presented as numbers and percentages, computed using the chi-square test to detect differences between the groups, while continuous variables were presented as mean ± standard deviation. A student’s *t*-test was used to detect differences between the groups. Factors with a *p*-value less than or equal to 0.25 in bivariate analysis were considered candidates for multivariate modeling. Multivariable logistic regression was performed to identify factors associated with hyperuricemia and increased hsCRP levels. Hosmer Lemeshow and pseudo-*R* square tests were checked, and no problems were found. The level of significance for statistical analysis was set at a *p*-value less than 0.05.

### Ethical consideration

Before data collection, ethical clearance was obtained from the Institutional Review Board of Jimma University with reference number of IHRPG1/5/2021. A permission letter had been obtained from the hospital, and data collection had started. Written informed consent was obtained from all patients before the interviews after providing information about the purpose and method of the study. Participants were coded to ensure confidentiality, and their information was kept confidential during data analysis. COVID-19 infection prevention measures recommended by the WHO were strictly followed during data collection to reduce the. The Declaration of Helsinki was strictly followed during the data collection.

## Results

### Sociodemographic characteristics

From a total of 172 participants, which is described in Table 1, 86 were DTG-treated and 86 were ATV/r-treated patients. The proportion of patients with an age greater than or equal to 40 was 52.3% (45/86) and 50% (43/86) among DTG and ATV/r-based regimens, respectively (*p* = 0.8). Regarding gender, 66.3% (57/86) and 69.8% (60/86) of them were female on DTG- and ATV/r-based regimens, respectively.

**Table 1:** Sociodemographic characteristics of study participants.

		Exposure category		
Variables	Category	DTG *n* (%)	ATV/r *n* (%)	*p*-value
Age	18–39	41 (47.7)	43 (50)	
	≥40	45 (52.3)	43 (50)	0.8
Gender	Male	29 (33.7)	26 (30.2)	
	Female	57 (66.3)	60 (69.8)	0.08
Religion	Muslim	25 (29.1)	29 (33.7)	
	Orthodox	28 (32.6)	27 (31.4)	0.8
	Others	33 (38.4)	30 (34.9)	
Ethnicity	Oromo	41 (47.7)	48 (55.8)	0.5
	Amhara	19 (22.1)	18 (20.9)	
	Others	26 (30.2)	20 (23.3)	
Marital status	Married	59 (68.6)	67 (77.9)	
	Unmarried	27 (31.4)	19 (22.1)	0.1
Educational status	Educated	81 (94.2)	80 (93)	
	Uneducated	5 (5.8)	6 (7)	0.8
Occupational status	Employed	26 (30.2)	21 (24.4)	
	Unemployed	60 (69.8)	65 (75.6)	0.4
Income (Birr/month)	<5,000	44 (51.2)	50 (58.1)	
	≥5,000	42 (48.8)	36 (41.9)	0.35
Residence	Urban	66 (76.7)	66 (76.7)	
	Rural	20 (23.3)	20 (23.3)	0.6
Smoking status	Yes	0 (0)	1 (1.2)	
	No	86 (100)	85 (98.8)	0.32
Alcohol use practice	Yes	40 (46.5)	47 (54.7)	
	No	46 (53.5)	39 (45.3)	0.28
Physical activity	Yes	58 (67.4)	68 (79.1)	
	No	28 (32.6)	18 (20.9)	0.8

DTG, dolutegravir; ATV/r, ritonavir-boosted atazanavir; HAART, highly active antiretroviral therapy.

### Clinical characteristics of the study participants

This study shows the proportion of patients with CD4 cell count <500 cells/mm^3^ was 36% (31/86) for DTG and 26.6% (22/86) for ATV/r-based regimen. Patients who had taken ART for more than 24 months were 58.1% (50/86) on DTG and 47.7% (41/86) on the ATV/r-based regimen. Furthermore, 34.9% (30/86) of DTG-treated and 23.3% (20/86) of ATV/r-treated patients had a BMI ≥ 25 kg/m^2^ (*p* = 0.093) (for more, see Table 2).

**Table 2:** Clinical characteristics of study participants.

		Exposure category		
Baseline variables	Category	DTG-based ART *n* (%)	ATV/r-based ART *n* (%)	*p*-value
CD4 cell count (cells/mm^3^)	≥500	55 (64)	64 (74.4)	
	<500	31 (36)	22 (26.6)	0.2
Viral load (copies/ml)	≤1,000	78 (90.7)	76 (88.4)	
	>1,000	8 (9.3)	10 (11.6)	0.6
Duration of HIV (years)	≤5	46 (53.5)	49 (57)	
	>5	40 (46.5)	37 (43)	0.6
Duration of HAART (Month)	≤24	36 (41.9)	45 (52.3)	
	>24	50 (58.1)	41 (47.7)	0.54
WHO clinical staging	I	21 (24.4)	25 (29.1)	
	II	33 (38.4)	26 (30.2)	
	III	32 (37.2)	35 (40.7)	0.5
	IV	0	0	
BMI (kg/m^2^)	<25	56 (65.1)	66 (76.7)	
	≥25	30 (34.9)	20 (23.3)	0.093
WC (cm)	< cut-of	43 (50)	42 (48.8)	
	≥ cut-of	43 (50)	44 (51.2)	0.9
WHR	< cut-of	41 (47.7)	44 (51.2)	
	≥ cut-of	45 (52.3)	42 (48.8)	0.6

NB, ATV/r, ritonavir-boosted atazanavir; BMI, body mass index; DTG, dolutegravir; HAART, highly active antiretroviral therapy; WC, waist circumference; WHR, waist-hip ratio.

### Mean difference among study participants

Table 3 shows higher mean levels of uric acid were observed among patients treated by DTG compared to ATV/r-based regimens (*p* = 0.02), but insignificant differences were observed regarding hsCRP levels (*p* = 0.18).

**Table 3:** Mean difference between study groups.

	Exposure category		
Variables	ATV/r-based regimen	DTG-based regimen	*p*-value
Uric acid (mean ± SD)	4.7 (1.7) mg/dl	5.38 (2.1) mg/dl	0.02
HsCRP (mean ± SD)	2.9 (2.8) mg/L	2.3 (2.5) mg/L	0.18

ATV/r, ritonavir-boosted atazanavir; DTG, dolutegravir; SD, standard deviation; hsCRP, high sensitivity CRP.

### Prevalence of hyperuricemia among study participants

**Figure 1:**
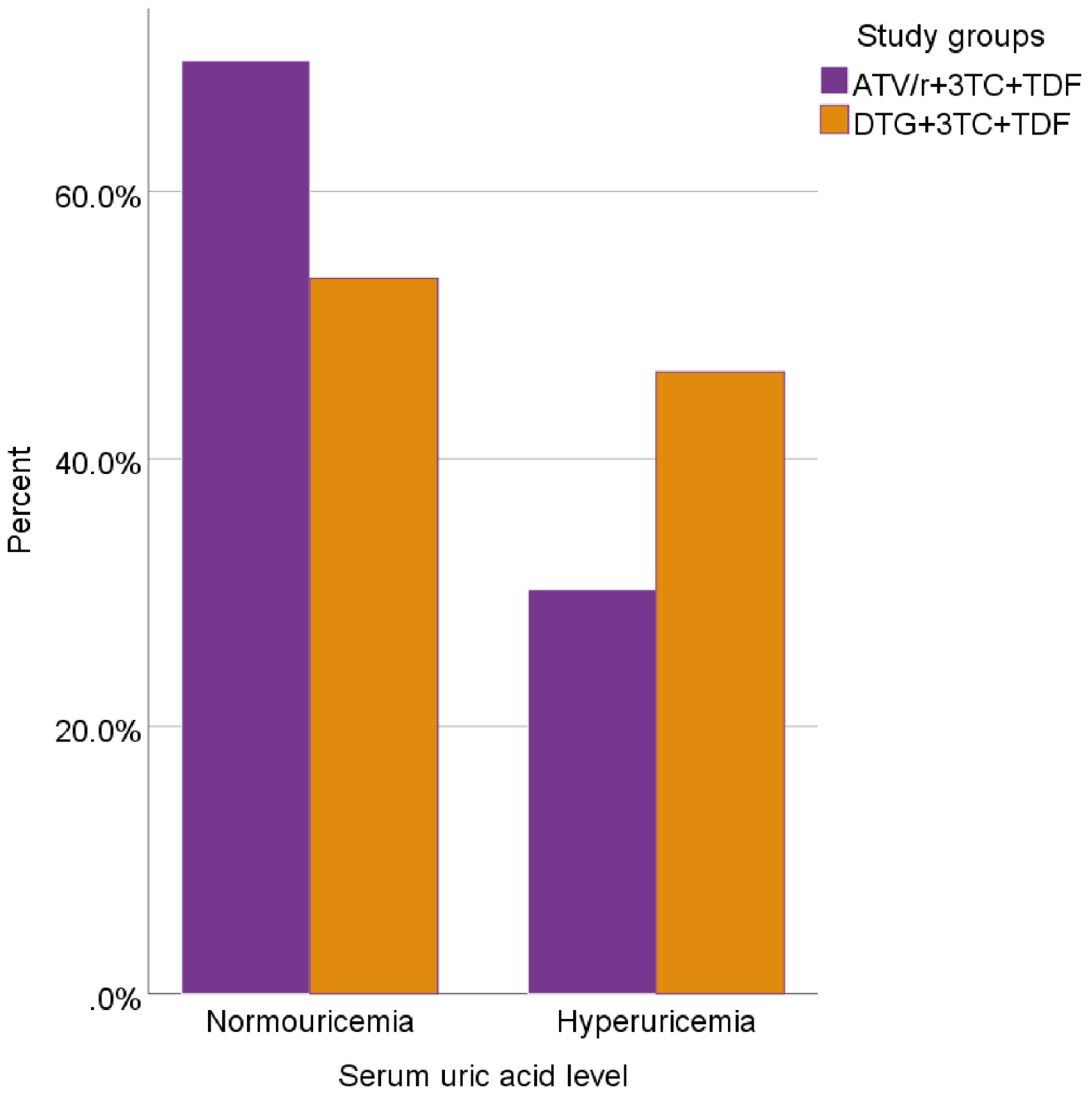
Prevalence of hyperuricemia among people living with HIV taking DTG and ATV/r-based ART.

The prevalence of hyperuricemia shown in Figure 1 was 46.5% (40/86) and 30.2% (26/86) among patients treated by DTG and ATV/r-based regimens of ART, respectively.

### The prevalence of hsCRP levels ≥2 mg/L

**Figure 2:**
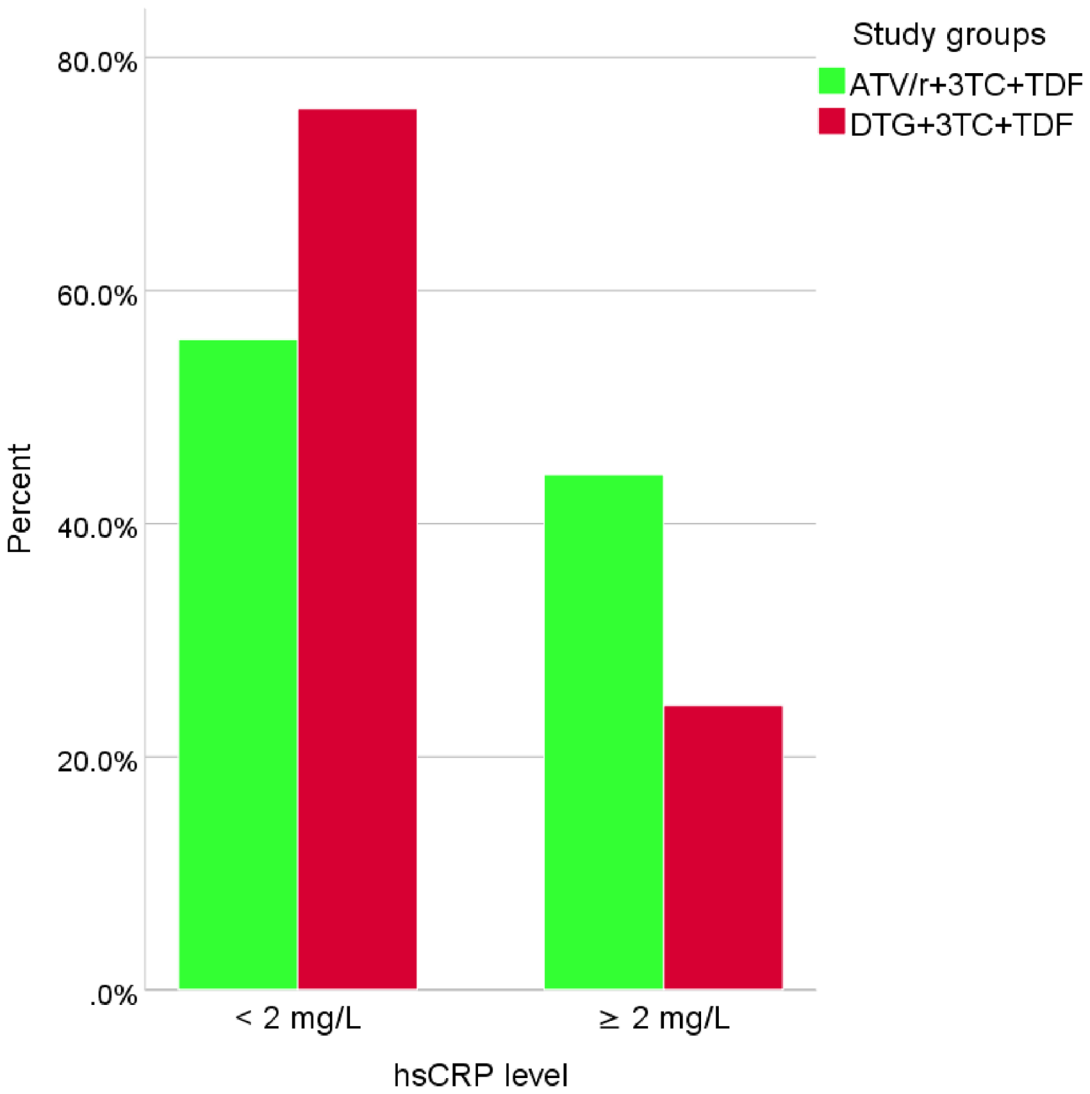
Prevalence of elevated hsCRP levels among people living with HIV taking DTG and ATV/r-based ART.

This study found (Figure 2) that 24.4% (21/86) of patients on the DTG-based regimen and 44.2% (38/86) on the ATV/r-based regimen have increased hsCRP levels ≥2 mg/L.

### Factors associated with hyperuricemia in people living with HIV on DTG and ATV/r-based ART

Multivariable logistic regression was performed for all predictor variables with *p* < 0.25 in the bivariate analysis (Table 4). In the multivariate analyses, the duration of ART and DTG-based ART were identified as significant predictors of hyperuricemia. It was observed that the odds of developing hyperuricemia were two times (AOR = 1.9; 95% CI: 1.04, 3.8; *p*-values = 0.04) more likely for patients treated with DTG than ATV/r-based regimen. Our study also revealed that patients living with HIV for more than 5 years had two times (AOR = 2; 95% CI: 1.2, 4.4; *p*-value = 0.02) more chance of having hyperuricemia than those living with HIV for less than or equal to 5 years.

**Table 4:** Factors associated with hyperuricemia among study participants.

		Hyperuricemia		Bivariate analysis		Multivariate analysis	
Variable	Category	Yes	No	COR (95% CI)	*p*-value	AOR (95%CI)	*p*-value
Age	18–39	28	56	1			
	≥40	38	50	1.5 (0.8, 2.8)	0.19	1.2 (0.6,2.4)	0.5
Gender	Female	41	76	1			
	Male	25	30	1.5 (0.8, 2.9)	0.19	1.7 (0.8,3.3)	0.2
Duration of ART	≤24	23	58	1			
(Months)	>24	43	48	2.3 (1.2, 4.3)	0.012	2 (1.2,4.4)	0.02
Duration of HIV	≤5	32	63	1			
(Years)	>5	34	43	1.6 (0.9,3)	0.11	1.5 (0.7,2.9)	0.3
Regimen types	ATV/r	26	60	1			
	DTG	40	46	2 (1.1, 3.8)	0.029	1.9 (1.04,3.8)	0.04
CD4 cell count	≥500	41	78	1			
(Cells/mm^3^)	<500	25	28	1.7 (0.9, 3.3)	0.115	1.3 (0.6,2.6)	0.5
BMI (kg/mm^2^)	<25	43	79	1			
	≥25	23	27	1.6 (0.8,3)	0.19	1.8 (0.8,3.3)	0.14
WC (cm)	Normal	27	58	1			
	Abnormal	39	48	1.7 (0.94,3.3)	0.079	1.8 (0.9,3.9)	0.08
WHR	<cut-of	37	48	1			
	≥cut-of	29	58	0.7 (0.35.1.2)	0.17	1.8 (0.9, 3.5)	0.06

NB: 1, reference category.

ATV/r, ritonavir-boosted atazanavir; BMI, body mass index; AOR, adjusted odd ratio; COR, crude odd ratio; CI, confidence interval; DTG, dolutegravir; HAART, highly active antiretroviral therapy; WC, waist circumference.

### Factors associated with elevated hsCRP levels among people living with HIV on DTG-based and ATV/r-based ART

Bivariate and multivariate analyses of the factors associated with elevated hsCRP levels are presented by the following (Table 5). All variables with *p* < 0.25 (age, duration of ART, HIV, BMI, WC, regimen type, and CD4 cell count) were included in the multivariate analysis. After adjusting for all these variables in multiple logistic regressions, ATV/r-based ART (AOR = 3; 95% CI: 1.5, 8.3; *p* = 0.002) and waist circumference (AOR = 2.5; 95% CI: 1.3, 5; *p* = 0.01) were identified as significant predictors of elevated hsCRP levels.

**Table 5:** Factors associated with increased hsCRP levels among study groups.

		hsCRP≥2 mg/L		Bivariate analysis		Multivariate analysis	
Variables	Category	Yes	No	COR (95% CI)	*p*-value	AOR (95%CI)	*p*-value
Age	18–39	25	59	1		1	
	≥40	34	54	1.5 (0.8, 2.8)	0.221	1.3 (0.7,2.7)	0.4
Duration of ART (months)	≤24	23	58	1		1	
	>24	36	55	1.7 (0.9, 3.1)	0.13	1.7 (0.9, 3.6)	0.2
Duration of HIV (years)	≤5	27	68	1			
	>5	32	45	1.7 (0.9, 3.3)	0.09	1.6 (0.8, 3.2)	0.2
Regimen types	DTG	21	65	1		1	
	ATV/r	38	48	2.5 (1.3, 4.7)	0.007	3 (1.5, 8.3)	0.002
CD4 cell count (cells/mm^3^)	≥500	36	83	1		1	
	<500	23	30	1.8 (0.9, 3.5)	0.09	1.6 (0.8, 3.4)	0.2
BMI (kg/m^2^)	<25	38	84	1		1	
	≥25	21	29	1.6 (0.85, 3.1)	0.15	1.9 (0.9, 4)	0.06
WC	Normal	20	65	1		1	
	Abnormal	39	48	2.6 (1.4, 5.1)	0.04	2.5 (1,3.5)	0.01

NB: 1, reference category. ATV/r, ritonavir-boosted atazanavir; BMI, body mass index; AOR, adjusted odd ratio; COR, crude odd ratio; CI, confidence interval; DTG, dolutegravir; ART, antiretroviral therapy; hsCRP, high-sensitivity C-reactive protein; WC, waist circumference.

## Discussion

This study explored the levels of uric acid and hsCRP as well as their associated factors among people living with HIV taking dolutegravir and ritonavir-boosted antiretroviral therapy. This is the first study in Ethiopia, given that the drug (DTG) is relatively new for HIV management in Ethiopia.

The prevalence of hyperuricemia among the whole participants was 76.7% (66/172), of which 46.5% (40/86) and 30.2% (26/86) were DTG-treated and ATV/r-treated patients, respectively (*p* = 0.028). It also found a higher mean level of uric acid among the patients treated by the DTG-based regimen than the ATV/r-based regimens. This observation is comparable to study findings from China (59), Portugal (27), and Japan (26) but is incongruent with another study finding reported from Japan (60). The proportion of treatment-naïve patients (71.6% in their study and with all were treatment-experienced in our study), male participants (91.9 and 51.1% in Japan and our study, respectively), in addition to study design, sociodemographic characteristics and differences in ethnicity might contribute to this incongruency of findings Although individuals of Africans origins had the lowest hyperuricemia and gout risk allele frequencies than Asian descent (61), they may respond differently to ART to result in hyperuricemia.

Consequently, according to our study findings, it was observed that the proportion of patients with increased hsCRP levels ≥2 mg/L was 24.4% (21/86) among patients with a DTG-based regimen and 44.2% (38/86) for patients treated with ATV/r-based regimen (*p* = 0.006), which was in line with San Francisco (52) and Boston (62). On the contrary, our finding is inconsistent with a study conducted in California (63). The possible reasons for this might be differences in sociodemographic, lifestyle, study design, ART combination, and ART exposure status. For instance, the study from California used TDF plus FTC as the backbone, which is TDF plus 3TC in our study. The participants were treatment-naïve in California and treatment-experienced in our study. In additions, the differences in race (which is whites in California and black Africans in our study) might contribute for this inconsistency of findings. For instance, the elevated levels of hsCRP were seen among African peoples compared to Europe and the United states (34). It is also essential to note that ART response of individuals might be varied due to variation in genetics that contributed for different plasma levels of hsCRP (64). Furthermore, our study was designed to assess factors associated with hyperuricemia and increased hsCRP levels. Accordingly, patients taking ART for more than 24 months were two times more likely to have hyperuricemia. This is similar to studies done in China and Nigeria (23, 65). Patients treated with a DTG-based regimen had a two-fold higher risk of developing hyperuricemia, which was in agreement with a study finding from China (59), which showed the number of patients with hyperuricemia increased with DTG-containing ART. ART-induced mitochondrial toxicity with increased lactate to compete with uric acid excretion by the renal tubules and increased cell turnover followed by increased degradation of nucleotides accompanied by elevated uric acid in the serum were some of the possibilities by which uric acid is elevated in people living with HIV on HAART (66–69). Patients with waist circumferences above the cutoff level had a two-fold increased chance of developing hsCRP levels ≥2 mg/L, which is in line with a study done in Brazil (70). This might be due to the fact that in obese patients, adipocytes produce powerful pro-inflammatory cytokines like tumor necrosis factor-α (TNF-α) and IL-6, which in turn stimulate the secretion of CRP in the liver and endothelial cells (71). Elevated hsCRP levels increased the risk of HIV patients on HAART for ASCVD, independent of traditional risk factors (33).

The ATV/r was associated with elevated hsCRP levels. This is comparable to study findings from Spain (40) and Italy (72). Ubiquitin-specific protease 18 (USP18) inhibitions lead to activation of nuclear factor kB (NF-kB) signaling, and augmented expression of pro-inflammatory cytokines with elevated inflammatory biomarkers is the possible way by which ATV/r increases the serum level of hsCRP levels (38).

### Limitations and strength

Concerning strength, this was the first study in Ethiopia that attempted to assess the effects of DTG and ATV/r-based regimens on serum uric acid and high-sensitivity C-reactive protein levels among people living with HIV, hence ultimately adding to the limited data. In spite of this strength, this study has several weaknesses. Since it is a cross-sectional study, we cannot associate causal relationships between the factors and outcomes under study. In addition, the study sample size was small; thus, it is difficult to generalize the findings to larger populations. Moreover, HIV-positive ART-naïve controls were not included, which may remove the effect of ART taken before starting DTG and ATV/r-based regimens of ART.

## Conclusion and recommendations

In general, our study showed a higher mean as well as proportion of uric acid with the use of a DTG-based regimen, whereas a higher proportion of hsCRP levels was found among those treated by ATV/r-based regimens. Being on a DTG-based regimen and taking HAART for a long period of time was significantly associated with hyperuricemia. Increased waist circumference and ATV/r-based regimen were identified as significant predictors associated with increased hsCRP levels. Therefore, it is important to consider and evaluate hsCRP levels for patients with increased waist circumference and treated by ATV/r containing ART, along with uric acid levels for patients on HAART for a long duration and taking DTG containing ART, to prevent the risk of having ASCVD. For this, policymakers should take these factors into account when developing public health initiatives on ART side effects management and when strengthening on going non-communicable disease reduction programs. We propose that researchers conduct a prospective cohort study with a larger sample size to determine the exact effects of DTG and ATV/r on serum uric acid and hsCRP levels. Lastly, we suggest a comparative analysis of DTG and ATV/r containing ART in ART-naïve, HIV-positive patients.

## Data availability statement

The original contributions presented in the study are included in the article/supplementary material, further inquiries can be directed to the corresponding authors.

## Ethics statement

The studies involving humans were approved by Institutional Review Board of Jimma University with reference number: IHRPG1/5/2021. The studies were conducted in accordance with the local legislation and institutional requirements. The participants provided their written informed consent to participate in this study.

## Author contributions

NW: Data curation, Methodology, Software, Visualization, Writing – original draft, Writing – review & editing, Conceptualization, Formal analysis, Investigation, Supervision. SN: Conceptualization, Investigation, Methodology, Software, Visualization, Writing – original draft, Writing – review & editing. RU: Formal analysis, Methodology, Software, Supervision, Visualization, Writing – original draft, Writing – review & editing. MJ: Data curation, Methodology, Software, Validation, Visualization, Writing – original draft, Writing – review & editing.
